# Prospects for the Role of Ferroptosis in Fluorosis

**DOI:** 10.3389/fphys.2021.773055

**Published:** 2021-12-07

**Authors:** Yi Zhang, Jialong Wu, Lai Jiang, Chenkang Lu, Zhengwei Huang, Bin Liu

**Affiliations:** ^1^Department of Endodontics and Operative Dentistry, Shanghai Ninth People’s Hospital, Shanghai Jiao Tong University School of Medicine, Shanghai, China; ^2^College of Stomatology, Shanghai Jiao Tong University, Shanghai, China; ^3^National Center for Stomatology, Shanghai, China; ^4^National Clinical Research Center for Oral Diseases, Shanghai, China; ^5^Shanghai Key Laboratory of Stomatology, Shanghai, China

**Keywords:** fluorosis, ferroptosis, oxidative stress, antioxidant enzymes, lipid peroxidation, iron metabolism

## Abstract

As a strong oxidant, fluorine can induce oxidative stress resulting in cellular damage. Ferroptosis is an iron-dependent type of cell death caused by unrestricted lipid peroxidation (LPO) and subsequent plasma membrane rupture. This article indicated a relationship between fluorosis and ferroptosis. Evidence of the depletion of glutathione (GSH) and increased oxidized GSH can be found in a variety of organisms in high fluorine environments. Studies have shown that high fluoride levels can reduce the antioxidant capacity of antioxidant enzymes, while increasing the contents of reactive oxygen species (ROS) and malondialdehyde (MDA), resulting in oxidative stress and fluoride-induced oxidative stress, which are related to iron metabolism disorders. Excessive fluorine causes insufficient GSH, glutathione peroxidase (GSH-Px) inhibition, and oxidative stress, resulting in ferroptosis, which may play an important role in the occurrence and development of fluorosis.

## Introduction

### Fluorosis

Fluoride is widely present in nature and has strong oxidizing properties. Excessive fluoride in nature poses a serious threat to the health of plants, animals, and humans. Animal studies have shown that fluoride is neurotoxic, leading to learning and memory impairment, as well as teratogenic effects that can induce malformations and premature aging of the reproductive system (Perumal et al., 2013). Excessive fluoride intake during human growth and development can cause a variety of health problems from mild effects on teeth and bones to severe kidney problems, neurotoxicity, and even cancer (Saeed et al., 2020). Moreover, the fluoride content in drinking water has been positively correlated with tooth and bone fluorosis (Mondal et al., 2016). The “Guidelines for Drinking-Water Quality” state that a fluoride concentration in drinking water greater than 1.5 mg/L increases the risk of dental fluorosis (WHO, 2017).

During the development of tooth enamel, excessive fluoride can inhibit the activity of alkaline phosphatase and induce apoptosis of ameloblasts through oxidative stress (Yang et al., 2015), which adversely affects the normal mineralization process of tooth enamel, resulting in chalky or brown plaques on the enamel, and in severe cases, substantial enamel defects. Dental fluorosis is more prevalent in areas, where fluorine-rich coal is burned, drinking water is polluted, and brick tea is consumed. A recent meta-analysis showed that since the defluorination of drinking water in China (fluorine content from 2.72 to 0.54 mg/L), the prevalence and severity of fluorosis among children has decreased significantly over the past two decades (Wang et al., 2021). In addition to fluoride intake, the risk of disease is also closely related to the susceptibility of individual genes. Polymorphisms of the calcitonin receptor, type I collagen α2 chain, and enamel matrix genes are thought to affect the occurrence of dental fluorosis (Jiang et al., 2015; Küchler et al., 2018).

#### Mechanisms of Fluorosis

Fluorine is a strong oxidant that can cause oxidative stress and subsequent cell damage. Studies have shown that within a certain period, the levels of intracellular reactive oxygen species (ROS) and apoptosis are linearly related to fluorine exposure (Ke et al., 2016). Fluoride causes lipid peroxidation (LPO), which destroys membrane phospholipids, resulting in extensive damage to intracellular organelles. In the endoplasmic reticulum of cells exposed to fluoride, the accumulation of unfolded and misfolded proteins in large quantities increases with fluoride concentrations, indicating that fluoride can induce the unfolded protein response and endoplasmic reticulum stress, which can trigger endoplasmic reticulum-mediated apoptosis (Liu et al., 2015).

Under physiological conditions, antioxidant cellular mechanisms regulate oxidative stress, but fluoride can inhibit gene expression and reduce the activity of superoxide dismutase (SOD; Wang et al., 2014). Due to the decreased expression and activity of antioxidant enzymes, the antioxidant capacity of cells is insufficient to resist the large amounts of ROS produced by fluoride, which results in cell damage, imbalance of cell redox homeostasis, and finally apoptosis.

Recent studies of the mechanisms underlying the onset of fluorosis have revealed that excessive fluorine can induce oxidative stress, regulate cellular redox homeostasis, and cause mitochondrial damage, endoplasmic reticulum stress, and altered gene expression (Zuo et al., 2018). This article proposes that ferroptosis may play a potential role in the occurrence and development of fluorosis, which can provide methods for the prevention and treatment of fluorosis.

### Ferroptosis

**Figure 1:**
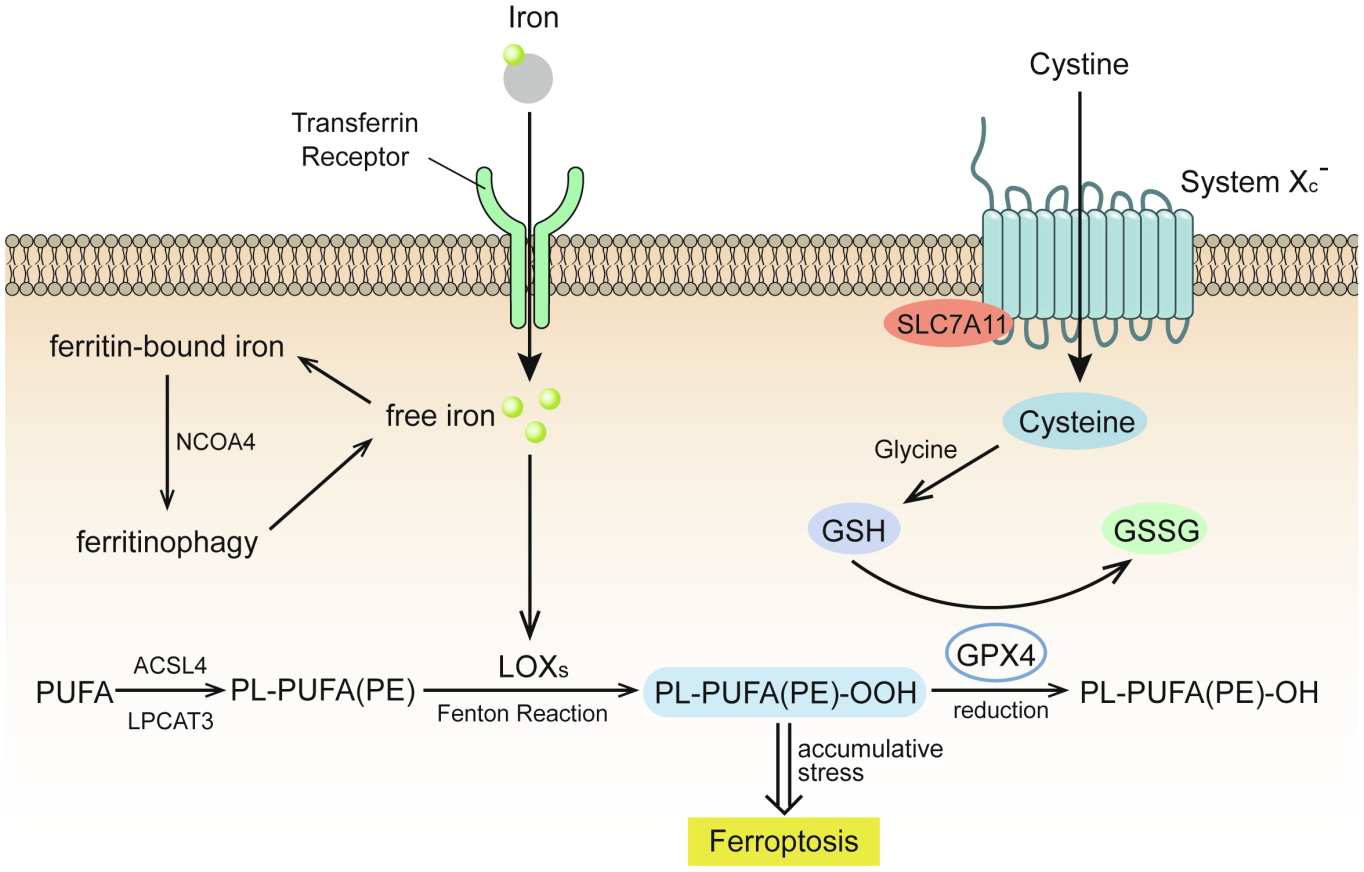
Glutathione (GSH) insufficient promotes the occurrence of ferroptosis (Stockwell et al., 2017). Extracellular cystine is exchanged with glutamate to enter the cell through the cystine/glutamate reverse transport system (system Xc-) and then reverts to cysteine. Under the catalysis of galactosidase and glutathione synthetase, cysteine and glycine co-synthesize glutathione (GSH), which is oxidized by glutathione peroxidase 4 (GPX4) to glutathione disulfide (GSSG), while GPX4 reduces toxic lipid peroxide to nontoxic lipid alcohol. Acyl-CoA synthetase long chain family member 4 (ACSL4) catalyzes the binding of free polyunsaturated fatty acids (PUFAs) to coenzyme A, and lysophosphatidylcholine acyltransferase 3 (LPCAT3) promotes their binding to membrane phospholipids to become PL-PUFAs (PE). Lipoxygenase (LOXs) mediates lipid peroxidation. PL-PUFA (PE) is oxidized to PL-PUFA (PE)-OOH, which becomes a ferroptosis signal. Extracellular iron ions enter the cell from the extracellular environment through transferrin and transferrin receptors, and generate reactive oxygen species (ROS) through the iron-dependent Fenton reaction or activate lipoxygenase to promote lipid peroxidation and promote ferroptosis.

Ferroptosis is an iron-dependent form of cell death caused by unrestricted LPO and subsequent plasma membrane rupture (Figure 1).

#### Amino Acid Metabolism

Extracellular cystine is exchanged with glutamate to enter the cell through the cystine/glutamate reverse transport system (system Xc-) and then reverts to cysteine. Under the catalysis of galactosidase and glutathione (GSH) synthetase, cysteine and glycine co-synthesize GSH, which is oxidized by glutathione peroxidase 4 (GPX4) to glutathione disulfide (Stockwell et al., 2017). GPX4 reduces toxic lipid peroxide (L-OOH) to nontoxic lipid alcohol (L-OH), which requires the catalytic selenocysteine residue of GPX4 and two electrons provided mainly by GSH (Jiang et al., 2021).

The lack of GSH leads to GPX4 inhibition, which causes cell damage due to excessive lipid peroxides, results in cell ferroptosis (Stockwell et al., 2017). The expression and activity of solute carrier family 7 member 11 and solute carrier family 3 member 2, two subunits of system Xc-, control the transport of cystine (Jiang et al., 2021). Excessive concentrations of extracellular glutamate will inhibit the transport of cystine, resulting in insufficient GSH synthesis.

#### Lipid Metabolism

Acyl-CoA synthetase long chain family member 4 (ACSL4) catalyzes the binding of free polyunsaturated fatty acids (PUFAs), such as arachidonic acid and adrenal acid, to coenzyme A, and lysophosphatidylcholine acyltransferase 3 (LPCAT3) promotes their binding to membrane phospholipids to become PL-PUFAs (PE). Arachidonate lipoxygenase (ALOX) mediates lipid peroxidation. Membrane electron transfer proteins participate in the production of ROS during lipid peroxidation. PL-PUFA (PE) is oxidized to PL-PUFA (PE)-OOH, which becomes a ferroptosis signal (Jiang et al., 2021). Reduced production of AA-PE membrane phospholipids inhibits lipid peroxidation and ferroptosis. For example, ACSL3 binds monounsaturated fatty acids to membrane phospholipids and 5' AMP-activated protein kinase (AMPK)-mediated phosphorylation of acetyl-CoA carboxylase alpha limits the production of PUFAs. Conversely, increasing the production of AA-PE membrane phospholipids promotes lipid peroxidation and ferroptosis. For example, as the activities of ACSL4, LPCAT3, and ALOX increase, the intake of PUFAs also increases, while AMPK-mediated phosphorylation of beclin1 inhibits GSH production (Chen et al., 2021).

#### Iron Metabolism

Extracellular iron ions enter the cell from the extracellular environment through transferrin and transferrin receptors. Upon recognition by nuclear receptor coactivator 4, intracellular ferritin is autophagocytosed and degraded by lysosomes to release free iron and generate ROS through the iron-dependent Fenton reaction or activate lipoxygenase to promote lipid peroxidation and promote ferroptosis. The Fenton reaction is a process by which excessive iron ions and hydrogen peroxide in the cell generate highly toxic hydroxyl radicals (Qian et al., 2019), which are than combined with the double bond of PL-PUFA to generate lipid peroxide (Tkachenko et al., 2021).

Drugs that increase iron-mediated toxicity, transferrin, and ferritin autophagy promote ferroptosis by increasing the intracellular content of free iron, while iron-chelating drugs, solute carrier family 40 member 1, exosomal-mediated ferritin export, mitochondrial protein-mediated iron-sulfur group biogenesis, and silencing of iron responsive element binding protein 2 have an opposite effect (Jiang et al., 2021).

## Relationship Between Fluorosis and Ferroptosis

**Figure 2:**
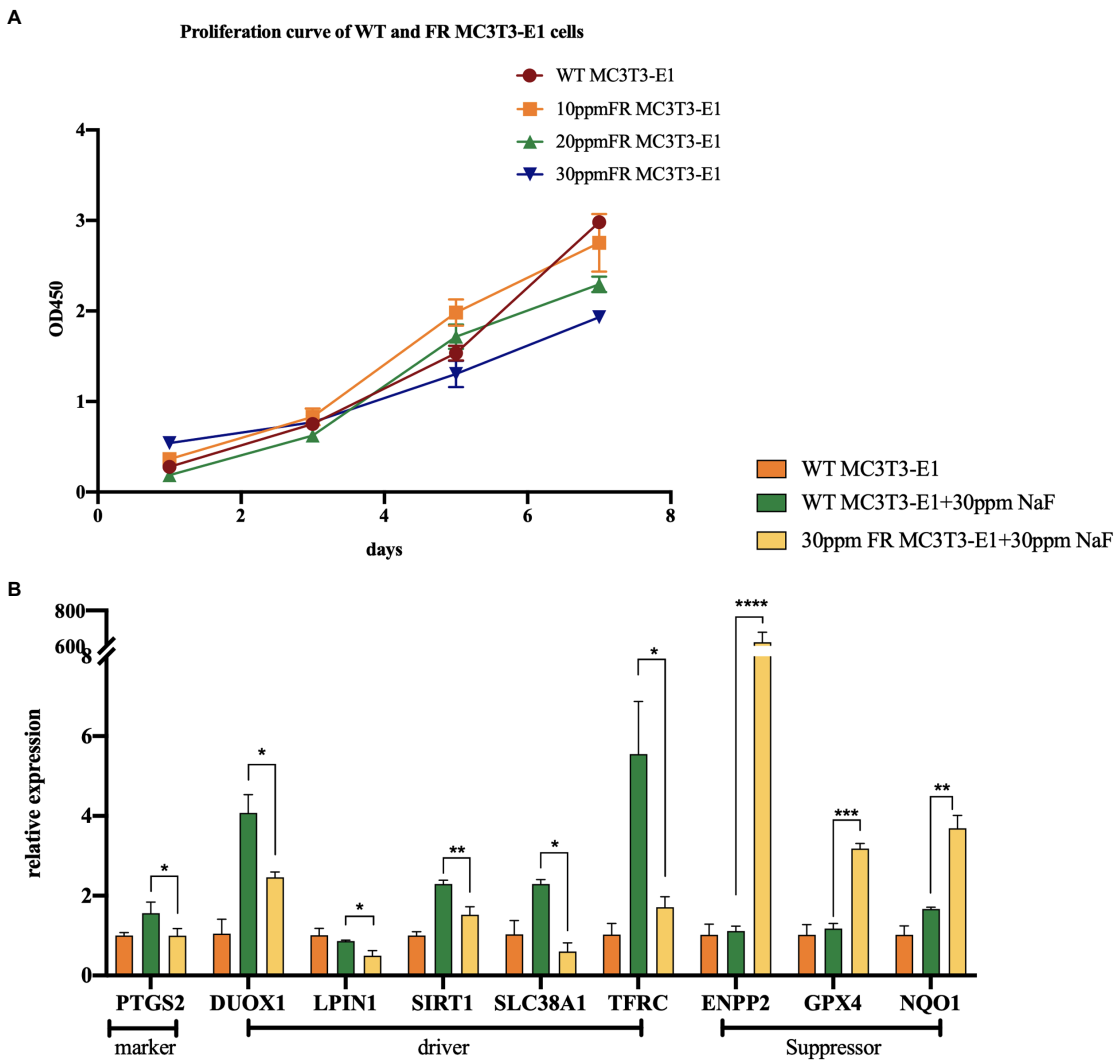
The changes of ferroptosis-related gene expression in fluoride-resistant MC3T3-E1 cells. **(A)** Cell-Counting-Kit-8 assay was used to determine the cell proliferation rate. Fluoride-resistant MC3T3-E1 (FR MC3T3-E1) cells, were inducted by sub-cultivating in 10 ppm fluoride concentration gradient ascending media, and continued till the proliferation rate of the cells in the fluoride media became normal. **(B)** Real-time PCR showed the changes of ferroptosis-related gene expression between 30 ppm FR MC3T3-E1 cells and WT MC3T3-E1 cells. When cultured in 30 ppm fluoride medium, the expression of Prostaglandin-Endoperoxide Synthase 2 (PTGS2) in FR cells was lower than that in WT cells, which is the marker of ferroptosis. Dual Oxidase 1 (DUOX1), Lipin 1 (LIPIN1), Sirtuin 1 (SIRT1), Solute Carrier Family 38 Member 1 (SCL38A1), and Transferrin Receptor (TFRC) are the drivers of ferroptosis. Ectonucleotide Pyrophosphatase/Phosphodiesterase 2 (ENPP2), GPX4, NAD(P)H Quinone Dehydrogenase 1 (NQO1) are ferroptosis suppressors. The result indicates that the expression of ferroptosis driver in FR cells is lower than that in WT cells. Similarly, the expression of ferroptosis suppressor in FR cells was significantly higher than that in WT cells. ^*^p < 0.05; ^**^p < 0.01; ^***^p < 0.001 and ^****^p < 0.0001.

Fluoride-resistant MC3T3-E1 (FR MC3T3-E1) cells, were inducted by sub-cultivating MC3T3-E1 cells (WT MC3T3-E1) in fluoride medium of 10 ppm fluoride concentration gradient ascending media, as mentioned in the previous article (Ran et al., 2017; Figure 2A). In order to explore the changes of ferroptosis-related gene expression in 30 ppm FR MC3T3-E1 cells, we performed real-time PCR (Figure 2B). The expression levels of these genes suggest that in the fluoride-containing medium, the ferroptosis degree in FR cells is lower than that in WT cells, indicating that there is a potential connection between cell fluorosis and ferroptosis. Although direct proof that excessive fluorine causes cell ferroptosis is lacking, the following studies can infer the relationship between fluorosis and ferroptosis.

### Fluorosis in a Variety of Organisms Shows Glutathione and Glutathione Peroxidase Depletion

Evidence of the depletion of GSH can be found in a variety of organisms in high fluorine environments, which is oxidized by glutathione peroxidase 4 (GPX4) to glutathione disulfide. As a result of GPX4 reduces L-OOH to L-OH (Stockwell et al., 2017), insufficient GSH is reported to inhibit the ability of GPX4 to reduce lipid peroxides, thereby promoting the occurrence of ferroptosis (Figure 1).

Perfluorooctane sulfonyl fluoride (PFOSF) is the precursor of many fluorine-containing compounds that are ubiquitous in the environment. Preliminary toxicological studies have confirmed that PFOSF can promote the oxidation of GSH to glutathione disulfide, which is then reduced to sulfinic acid (Jin et al., 2020).

Digital gene expression, gene ontology, and the Kyoto Encyclopedia of Genes and Genomes analysis of the transcriptome profile of the silkworm testis treated with NaF showed that many differentially expressed genes are involved in the stress response and oxidative phosphorylation. In the NaF treatment group, the activity of glutathione S-transferase increased, while the content of GSH decreased (Tang et al., 2018). At a concentration of 10 mg/L in water, fluoride can induce antioxidant responses of aquatic plants, while excessive fluoride (20–40 mg/L) results in unbalanced metabolism and oxidative damage (Gao et al., 2018). Excessive fluoride concentrations in water have detrimental effects on liver and kidney function, and ovarian development in fish, while reducing the production of reproductive hormones and eggs. There is a positive correlation between the hepatocyte apoptosis index and fluoride levels. Fluoride-induced oxidative stress in fish manifests as an increase in lipid peroxidation and consumption of GSH levels. The activity and mRNA expression levels of antioxidant enzymes, such as SOD and glutathione peroxidase (GSH-Px), significantly decreased, while the content of malondialdehyde (MDA), the end product of lipid peroxidation, significantly increased (Cao et al., 2013, 2015; Li et al., 2020).

Similar conclusions have been obtained in mammals. Excessive fluoride intake can cause oxidative stress and damage the antioxidant system in chickens. It reduced the activities of SOD and GSH-Px, increased ROS and MDA (Chen et al., 2011; Miao et al., 2017; Wang et al., 2018). Research on the antioxidant status of buffalo calves found that NaF exposure led to increased oxidative stress and a significant increase in LPO with significant reductions in GSH-Px and catalase (CAT) activities and GSH levels (Gill and Dumka, 2016).

Excessive sodium fluoride can cause pathological changes in the morphologies of the rat liver and lung, as well as increase serum levels of aspartate aminotransferase, alanine aminotransferase, and MDA, while decreasing total antioxidant capacity (T-AOC) and serum levels of SOD and GSH-Px (Mohammed et al., 2017; Mittal et al., 2018; Li et al., 2021). It has been reported that exposure to 10 ppm of fluoride will significantly increase serum level of ROS in rats, while reducing serum levels of GSH. Interestingly, exposure to fluorine at 50 and 100 ppm will not produce more obvious toxicity, thus the underlying mechanism of action is unclear (Chouhan and Flora, 2008). Similarly, excessive intake of fluoride can reduce the developmental potential of mouse oocytes by inducing oxidative stress and apoptosis. The depletion of antioxidant enzymes, such as GSH, glucose 6 phosphate dehydrogenase, SOD, and GSH-Px, with an increase in nitric oxide synthase 2 was detected in the mouse liver (Zhou et al., 2015; Wang et al., 2017). As compared to the brain, the liver is more sensitive to the toxic effects of fluoride. Interestingly, the combined exposure of arsenic and fluoride causes less obvious toxicity, and the joint exposure of these two toxic substances does not cause synergistic toxicity. In some cases, joint exposure to arsenic and fluoride will have an antagonistic effect (Mittal and Flora, 2006). Cytology experiments have found that NaF at concentrations of 5–20 mg/L may cause central nervous system poisoning by stimulating the conversion of BV-2 (mouse microglia) cells into activated microglia. In BV-2 cells treated with fluoride, SOD levels decreased, while those of MDA, ROS, superoxide anions (O^2−^), and NOS significantly increased in a dose-dependent manner (Shuhua et al., 2012).

It is found that in southwestern China, the people with endemic fluorosis had significantly reduced the levels of antioxidant enzyme activity including copper/zinc SOD, GSH-Px, and CAT, while those exposed to higher levels of fluorine had significantly lower serum antioxidant enzyme activities (Wang et al., 2014). Cytological studies found that exposure to 0.1 and 1% ammonium hexafluorosilicate (SiF) for 10 and 30 min caused a sharp increase in the cellular GSH consumption of human gingival fibroblasts (Song et al., 2013). Studies on human hepatocytes (L-02) have found that fluoride causes an increase in the LPO content of L-02 cells and a decrease in GSH content, with a significant dose-effect relationship (Wang et al., 2004).

### Fluorosis and Lipid Peroxidation

Aldehydes, such as MDA, as the final product of lipid peroxidation, produce toxicity by changing the structure and function of nucleic acids and proteins, and thus are considered as the “execution” stage of ferroptosis (Gaschler and Stockwell, 2017). The level of Thiobarbituric Acid Reactive Substances (TBARS) test represents the level of MDA, which is used to measure the levels of lipid peroxidation. The TBARS levels in the kidneys, lungs, and testes of rats exposed to 30 mg/L of NaF throughout pregnancy and lactation were higher than those in the control group, and significant tissue destruction was observed (Karaoz et al., 2004; Oncu et al., 2006, 2007). The TBARS levels in blood samples of Ukrainian children with chronic fluorosis (exposed to drinking water fluoride at >1.5 ppm for >5 years) in endemic areas were higher than those of healthy children living in non-fluorosis areas, indicating that fluorosis increased lipid peroxidation (Tkachenko et al., 2021).

Dietary fluoride supplementation leads to decrease antioxidant enzyme activities in the pig liver, in addition to decreased abilities to scavenge free radicals and excessive production of LPO (Zhan et al., 2006). Mice exposed to fluoride (NaF) at 15 ppm for 8 months showed increased consumption of GSH, lipid peroxidation, and catalase activity in the liver and brain. Fluorine-induced DNA damage and damaged DNA repair mechanisms trigger liver and brain cell apoptosis and tissue structure damage (Bhowmik et al., 2020). Therefore, high fluoride levels can reduce the activities of antioxidant enzymes, including SOD, CAT and GSH-Px, while simultaneously increasing ROS and MDA contents, resulting in oxidative stress.

### Fluorosis and Iron Metabolism

Extracellular iron ions enter the cell from the extracellular environment through transferrin and transferrin receptors. Upon recognition by nuclear receptor coactivator 4, intracellular ferritin is autophagocytosed and degraded by lysosomes to release free iron and generate ROS through the iron-dependent Fenton reaction or activate lipoxygenase to promote lipid peroxidation and promote ferroptosis (D’Herde and Krysko, 2017).

Fluorosis can significantly change mineral metabolism. For example, the kidney tissue of sheep in areas with endemic fluorosis reportedly had significantly higher iron concentrations (Çetin and Yur, 2016). Fluoride-induced oxidative stress has been correlated to iron metabolism disorders, which are characterized by increased iron and hepcidin levels, but decreased expression of iron transporters (Niu et al., 2018). *In vitro* experiments found that human red blood cells treated with NaF produced methemoglobin and oxyhemoglobin, with increased heme degradation, resulting in the release of free iron from porphyrin rings (Maheshwari et al., 2021). These findings suggested that fluoride exposure may have a potential impact on iron metabolism.

## Perspectives and Conclusion

It is generally believed that the mechanism of fluorosis mainly revolves around oxidative stress, enzyme and protein inactivation, acid-base balance, and electrolyte metabolism disorders. However, excessive fluorine may cause a decrease in GSH with a simultaneous decrease the activity of antioxidant enzymes, such as SOD and GSH-Px, while the content of MDA, the end product of lipid peroxidation, significantly increase. They all indicate an increase in ferroptosis. Our study result indicated that ferroptosis may play an important role in the occurrence of fluorosis. However, it just a preliminary experiment based *in vitro*. Further researches are needed to determine the role of ferroptosis in fluorosis. Secondly, it is necessary to determine the pathway of ferroptosis participate in the occurrence and development of fluorosis, and then to determine the relationship between fluoride and these pathways. These studies will provide solutions for the prevention and treatment of fluorosis.

## Data Availability Statement

The raw data supporting the conclusions of this article will be made available by the authors, without undue reservation.

## Author Contributions

YZ, JW, LJ, and CL collected and analyzed the data. YZ drafted the article. BL and ZH revised the article. All authors contributed to the article and approved the submitted version.

## Funding

This work was sponsored by Natural Science Foundation of Shanghai (grant No. 21ZR1455700) and the Research Discipline fund (No. KQYJXK2020) from Ninth People’s Hospital, Shanghai Jiao Tong University School of Medicine, and College of Stomatology, Shanghai Jiao Tong University.

## Conflict of Interest

The authors declare that the research was conducted in the absence of any commercial or financial relationships that could be construed as a potential conflict of interest.

## Publisher’s Note

All claims expressed in this article are solely those of the authors and do not necessarily represent those of their affiliated organizations, or those of the publisher, the editors and the reviewers. Any product that may be evaluated in this article, or claim that may be made by its manufacturer, is not guaranteed or endorsed by the publisher.
